# Epidemics to eradication: the modern history of poliomyelitis

**DOI:** 10.1186/1743-422X-4-70

**Published:** 2007-07-10

**Authors:** Nidia H De Jesus

**Affiliations:** 1 Department of Molecular Genetics & Microbiology, Stony Brook University School of Medicine, Stony Brook, New York, USA

## Abstract

Poliomyelitis has afflicted humankind since antiquity, and for nearly a century now, we have known the causative agent, poliovirus. This pathogen is an enterovirus that in recent history has been the source of a great deal of human suffering. Although comparatively small, its genome is packed with sufficient information to make it a formidable pathogen. In the last 20 years the Global Polio Eradication Initiative has proven successful in greatly diminishing the number of cases worldwide but has encountered obstacles in its path which have made halting the transmission of wild polioviruses a practical impossibility. As we begin to realize that a change in strategy may be crucial in achieving success in this venture, it is imperative that we critically evaluate what is known about the molecular biology of this pathogen and the intricacies of its interaction with its host so that in future attempts we may better equipped to more effectively combat this important human pathogen.

## Background

The word *poliomyelitis*, the medical term used to describe the effect of poliovirus (PV) on the spinal cord, is derived from the Greek words for gray (*polio*) and marrow (*myelon*). The first known clinical description of poliomyelitis is attributed to Michael Underwood, a British physician, who in 1789 reported observing an illness which appeared to target primarily children and left those afflicted with residual debility of the lower extremities. In subsequent years, additional cases of poliomyelitis would be reported. Initial outbreaks in Europe were documented in the early 19^th ^century and outbreaks in the United States were first reported in 1843. However, it was not until the early 20^th ^century that the number of paralytic poliomyelitis cases reached epidemic proportions.

In 1938, in efforts to support care for patients with poliomyelitis as well as fund research to combat the illness, the National Foundation for Infantile Paralysis (now the March of Dimes) was established. The number of paralytic cases in the United States, estimated to have been in excess of 21,000, peaked in 1952. Fortunately, on April 12, 1955, the March of Dimes declared that the Salk polio vaccine was both safe and effective. Then, in 1963, the development of a second vaccine, the Sabin polio vaccine, was announced. With the introduction of effective vaccines, the incidence of poliomyelitis rapidly declined. Indeed, in the United States, the last case of poliomyelitis due to infection with wild type (*wt*) virus was reported in 1979. Less than a decade later, in 1988, the World Health Organization (WHO) launched a global campaign to eradicate PV.

Since initial descriptions of poliomyelitis were first documented to the present time, innumerable milestones have been reached in understanding the molecular biology of PV and the pathogenesis of poliomyelitis. Such advances have certainly led to the more effective management of poliomyelitis. Nonetheless, many questions remain unanswered. One such question pertains to the determinants of neuropathogenesis, specifically regions of the virus genome important for aspects of virus replication in the cells which it targets.

In this review, the current state of our understanding of the molecular biology and pathogenesis of poliovirus, as it relates to current eradication efforts, is explored.

### Poliovirus classification

PV was discovered to be the causative agent of poliomyelitis in 1909 by Karl Landsteiner and Erwin Popper, two Austrian physicians [109]. Owing to the expression of three unique sets of four different neutralization antigenic determinants on the poliovirion surface referred to as N-Ag1, 2, 3A, and 3B [110,155], the virus occurs in three serotypes, termed types 1, 2, and 3, where the names Mahoney, Lansing, and Leon designate a strain of each serotype, respectively [21,98,125,137]. The polioviruses are classified as members of the *Picornaviridae*, a large family of small RNA viruses, consisting of nine genera: *Enterovirus*, *Rhinovirus*, *Cardiovirus*, *Aphthovirus*, *Hepatovirus*, *Parechovirus*, *Erbovirus*, *Kobuvirus*, and *Teschovirus *(Table 1). The *Enterovirus *genus, to which the polioviruses belong, can be further subdivided into eight clusters (i.e., *Poliovirus*, *Human enterovirus A*, *Human enterovirus B*, *Human enterovirus C*, *Human enterovirus D*, *Simian enterovirus A*, *Bovine enterovirus*, and *Porcine enterovirus B*) (Table 2), which include predominantly human pathogens exhibiting marked variation in the disease syndromes they produce.

**Table 1:** Classification within the *Picornaviridae*

**Genus**	**Type Species**	**Serotypes**
*Enterovirus*	*Poliovirus*	3
	*Human enterovirus A*	17
	*Human enterovirus B*	56
	*Human enterovirus C*	13
	*Human enterovirus D*	3
	*Simian enterovirus A*	1
	*Bovine enterovirus*	2
	*Porcine enterovirus B*	2
*Rhinovirus*	*Human rhinovirus A*	74
	*Human rhinovirus B*	25
*Cardiovirus*	*Encephalomyocarditis virus*	1
	*Theilovirus*	3
*Aphtovirus*	*Foot-and-mouth disease virus*	7
	*Equine rhinitis A virus*	1
*Hepatovirus*	*Hepatitis A virus*	1
	Avian encephalomyelitis-like virus	1
*Parechovirus*	*Human parechovirus*	3
	*Ljungan virus*	2
*Erbovirus*	*Equine rhinitis B virus*	2
*Kobuvirus*	*Aichi virus*	1
*Teschovirus*	*Bovine kobuvirus*	1
	*Porcine teschovirus*	11

**Table 2:** Classification within the *Enterovirus *Genus

**Clusters**	**Serotypes**	**Receptors**
*Poliovirus*	poliovirus 1 (PV1), PV2, PV3	CD155 ^[122]^
*Human enterovirus A*	coxsackievirus A2(CV-A2) - CV-A8, CV-A10, CV-A12, CV-A14, CV-A16	
	enterovirus 71 (EV-71), EV-76, EV-89 - EV-92	
*Human enterovirus B*	coxsackievirus B1 (CV-B1) - CV-B6	CAR,^[13] ^DAF^[12]^
	CV-A9	α_v_β_3 _integrin^[169]^
	echovirus 1 (E-1) - E-7, E-9, E-11 - E-21, E-24 - E-27, E-29 - E-33	
	EV-69, EV-73 - EV-75, EV-77 - EV-88, EV-93, EV-97, EV-98, EV-100, EV-101	
*Human enterovirus C*	CV-A1, CV-A11, CV-A13, CV-A17, CV-A19, CV-A22, CV-A24,	ICAM-1 (CV-A21^[176]^)
	EV-95, EV-96, EV-99, EV-102	
*Human enterovirus D*	EV-68, EV-70, EV-94	
*Simian enterovirus A*	simian enterovirus A1 (SEV-A1)	
*Bovine enterovirus*	bovine enterovirus 1 (BEV-1), BEV-2	
*Porcine enterovirus B*	porcine enterovirus 9 (PEV-9), PEV-10	

Admittedly, the initial classification of human enteroviruses was based on the clinical manifestations observed in human infections as well as on the pathogenesis in intracranially- and subcutaneously-inoculated experimental suckling mice. The four categories into which human enteroviruses were subdivided were: (1) polioviruses, which caused flaccid paralysis (poliomyelitis) in humans but not in suckling mice lacking CD155; (2) coxsackie A viruses (CAV), which were linked to human central nervous system (CNS) pathology and skeletal muscle inflammation (myositis) as well as acute flaccid paralysis in suckling mice; (3) coxsackie B viruses (CBV), associated with ailments of the human cardiac and central nervous systems, and necrosis of the fat pads between the shoulders, focal lesions in skeletal muscle, brain, and spinal cord, as well as spastic paralysis in the suckling mouse experimental model; and (4) echoviruses, which were not originally associated with human disease nor with paralysis in mice [41,121,201]. With groundbreaking advances in molecular biology, a modified classification stratagem has evolved. Under the new scheme, human enteroviruses are subdivided into five species: *Poliovirus *and *Human enterovirus A*, *B*, *C*, and *D*. The three PV serotypes (i.e., PV1, 2, and 3) constitute the species *Poliovirus*, and 11 coxsackie A virus serotypes (i.e., CAV1, 11, 13, 15, 17, 18, 19, 20, 21, 22, and 24) constitute the *Human enterovirus C *(HEV-C) [96] (Table 2). But recently, the Executive Committee of the International Committee on Taxonomy of Viruses (ICTV) has endorsed a proposal, which awaits ratification by the ICTV membership, to move the polioviruses into the *Human enterovirus C *species. On the basis of genome sequences, the C-cluster human enteroviruses bearing the greatest degree of relatedness to the polioviruses are CAV11, CAV17, and CAV20 [31]. Indeed, genetically, these three C-cluster coxsackie A viruses differ notably from the polioviruses only in the structural (P1) capsid region [31].

### The poliovirus genome

**Figure 1:**
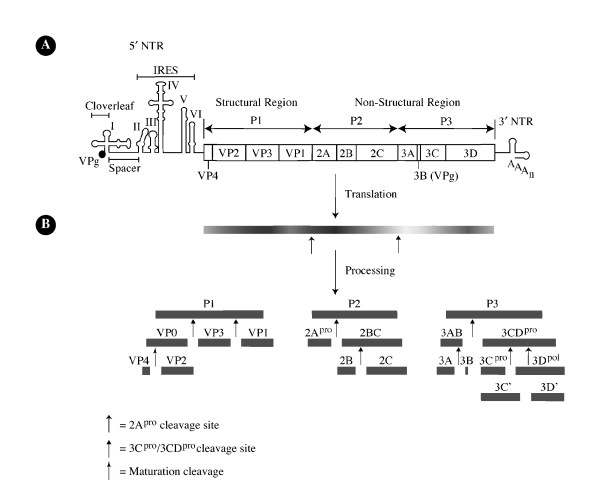
Genomic structure of poliovirus type 1 (Mahoney) [PV1(M)] and proteolytic processing of its polyprotein. (A) The PV genome consists of a single-stranded, positive-sense polarity RNA molecule, which encodes a single polyprotein. The 5' non-translated region (NTR) harbors two functional domains, the cloverleaf and the internal ribosome entry site (IRES), and is covalently linked to the viral protein VPg. The 3'NTR is poly-adenylated. (B) The polyprotein contains (N terminus to C terminus) structural (P1) and non-structural (P2 and P3) proteins that are released from the polypeptide chain by proteolytic processing mediated by virally-encoded proteinases 2A^pro ^and 3C^pro^/3CD^pro ^to ultimately generate eleven mature viral proteins [197]. Three intermediate products of processing (2BC, 3CD, and 3AB) exhibit functions distinct from those of their respective final cleavage products.

The genome of the polioviruses as well as that of members of the *Human enterovirus C *cluster is approximately 7400 nucleotides (nt) in length (PV, 7441 nt) and composed of single-stranded RNA consisting of three distinct regions: a relatively long 5'NTR (PV, 742 nt) that is covalently linked to the virus-encoded 22-amino acid long VPg protein [110,196]; a single open reading frame (ORF) encoding the viral polyprotein; and a comparatively short 3'NTR followed by a virus-encoded poly(A) tract of variable length (PV, 60 adenine residues) [47,97,163,182,202] (Fig. 1A).

**Figure 2:**
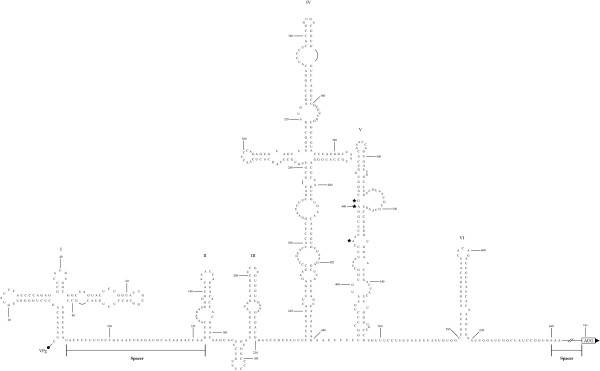
Secondary structure of the PV1(M) 5'NTR. This genomic region has been divided into six domains (I to VI) [197], of which domain I constitutes the cloverleaf and the remaining domains (II to VI) comprise the IRES. Spacer sequences without complex secondary structure exist between the cloverleaf and the IRES (nt 89–123) and between the IRES and the initiation codon (nt 620–742). Mutations in the 5'NTR of the Sabin PV type 1, 2, and 3 vaccine strains localizing to nucleotides 480 (A to G) [94], 481 (A to G) [129], and 472 (C to U) [194], respectively, each denoted by a star, confer attenuation in the CNS and deficient replication in neuroblastoma cells [106, 107] as well as reduced viral RNA translation efficiency [184-186].

The 5'NTR is predicted to harbor a significant degree of complex secondary structure [1,158,179] (Fig. 2). Computer analysis has predicted six domains (domains I-VI) within the 5'NTR, many of which have been validated by genetic and biochemical analyses [53] as well as visualized by electron microscopy [10]. In this region of the genome, eight cryptic AUG triplets have been identified which precede the initiation codon at nt 743. This segment of the genome can be further subdivided into: (i) the 5'-terminal cloverleaf, an indispensable *cis*-acting element in viral RNA replication [3,113,144,147] as well as in regulating the initiation of translation; and (ii) the IRES [197], which mediates cap-independent translation of the viral mRNA by facilitating initiation of translation independent of a capping group and even a free 5' end [36,90,91,147,149,150].

In contrast to the 5'NTR, comparatively less is known about the 3'NTR. Nonetheless, this region is known to be poly-adenylated and predicted to exhibit conserved secondary structures consisting of two hairpins [89,160]. Moreover, evidence indicates that it has a functional role in RNA replication [31,32,50,89,108,123,157,159,160]. Specifically, it has been shown that while deletion of the 3'NTR has only minimal effects on the ability of PV to propagate in HeLa cells, the ability of the virus to propagate in cells of neuronal origin is markedly reduced both *in vitro *and *in vivo *[31].

The 250-kDa polyprotein encoded by the single ORF can be further subdivided into regions denoted P1, P2, and P3, encoding the structural and nonstructural proteins. Following translation of pUp-terminated mRNA [81,134], proteolytic cleavage of the unstable "polyprotein" by virus-encoded proteinases, 2A^pro ^and 3C/3CD^pro ^in *cis *and in *trans *[78] (Fig. 1B), gives rise to proteins with functions in viral proliferation. Processing of the polyprotein is thought to proceed in accordance to a pathway established by protein folding resulting in masking of certain cleavage sites and by amino acid sequences adjoining the scissile bond [78] The first cleavage of the genomic polyprotein at a tyrosine-glycine dipeptide is catalyzed by the 2A proteinase and results in release of a 97-kDa polyprotein consisting of the P1 structural segment of the genome [190]. Subsequent cleavages of the P1 precursor into stable end products VP0, VP3, and VP1 is mediated by the 3CD proteinase [203]. Cleavage of VPO into capsid proteins VP4 and VP2 occurs during maturation of the virion and is mediated by an unknown mechanism that has been hypothesized to be viral proteinase independent [77]. The cleavage of P2 and P3 precursors into stable end products [2A^pro^, 2B, 2BC, 2C, 3A, 3AB, 3B (VPg), 3C/3CD^pro^, and 3D^pol^] at glutamine-glycine dipeptides is catalyzed by the 3C^pro^/3CD^pro ^[76].

### The cellular life cycle of poliovirus

**Figure 3:**
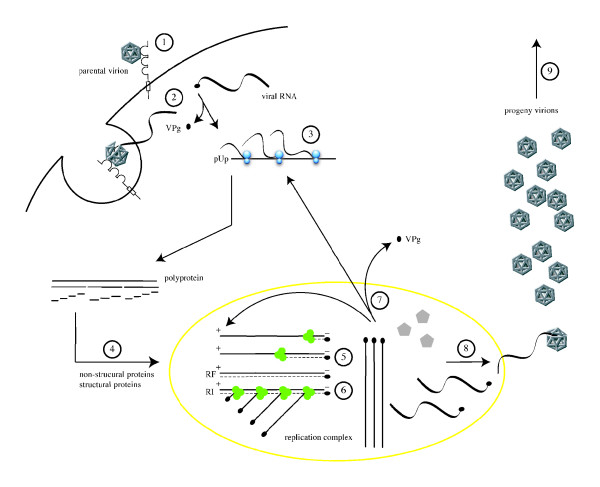
The cellular life cycle of poliovirus. It is initiated by binding of a poliovirion to the cell surface macromolecule CD155, which functions as the receptor (1). Uncoating of the viral RNA is mediated by receptor-dependent destabilization of the virus capsid (2). Cleavage of the viral protein VPg is performed by a cellular phosphodiesterase, and translation of the viral RNA occurs by a cap-independent (IRES-mediated) mechanism (3). Proteolytic processing of the viral polyprotein yields mature structural and non-structural proteins (4). The positive-sense RNA serves as template for complementary negative-strand synthesis, thereby producing a double-stranded RNA (replicative form, RF) (5). Initiation of many positive strands from a single negative strand produces the partially single-stranded replicative intermediate (RI) (6). The newly synthesized positive-sense RNA molecules can serve as templates for translation (7) or associate with capsid precursors to undergo encapsidation and induce the maturation cleavage of VP0 (8), which ultimately generates progeny virions. Lysis of the infected cell results in release of infectious progeny virions (9).

The life cycle of PV occurs within the confines of the cytoplasm of infected cells (Fig. 3). It is initiated by attachment of the poliovirion to the N-terminal V-type immunoglobulin-like domain of its cell surface receptor, the human PV receptor (hPVR) or CD155 [99,122,175]. Release of the virus RNA into the cell cytoplasm (uncoating) is thought to occur by destabilization of the virus capsid secondary to CD155-mediated release of the myristoylated capsid protein VP4 and of the putative N-terminal amphipathic helix of VP1 from deep within the virion [reviewed in [84]]. Subsequently, the myristoylated VP4 and VP1 amphiphatic helix are thought to insert into the cell membrane [58], thereby leading to the creation of pores in the cell membrane through which the virus RNA may enter the cytoplasm. Alternatively, since the virus can be found on endosomes [101,102,139], others believe the virus is taken up by receptor-mediated endocytosis. However, both classic endocytotic pathways (clathrin-coated pits or caveoli) as the means of uptake have been excluded [45,84]. Additionally, if entry of the virus involves endosomes, acidification of this compartment is not necessary for release of the virus RNA into the cytoplasm [70]. Thus the exact mechanism by which the virus releases its RNA genome into the cytoplasm of infected cells remains to be elucidated.

Nonetheless, once in the cytoplasm of infected cells, an unknown cellular phosphodiesterase is believed to cleave the 5'NTR-linked viral protein VPg. This process is followed by initiation of translation of the RNA genome by host cell ribosomes [196]. Concurrently, shut off of cap-dependent host cell translation occurs by 2A^pro^-mediated cleavage of the eukaryotic translation initiation factor 4G (eIF4G), an element of the cap recognizing complex eIF4F [100,181,193]. Interestingly, a byproduct of eIF4G cleavage binds viral RNA and promotes IRES-dependent translation of the viral polyprotein [140]. Moreover, inhibition of host cell transcription occurs via inactivation of transcription factor TFIIIC [40] and cleavage of the TATA box binding protein (TBP) by 3C^pro ^[199].

With the synthesis of virus proteins, replication of the RNA begins. Initially, for the first three hours following infection of a permissive host cell, the kinetics of RNA replication is exponential. This is followed by a linear phase for one and a half hours, which ultimately enters a period of rapid decay in the rate of synthesis [172]. The process of RNA replication takes place in the cytoplasm on host cell endoplasmic reticulum-derived rosette-like membranous structures, the formation of which is induced by viral proteins 2C and 2BC [14,38,188]. Subsequently, a hydrophobic domain in 3AB anchors this protein in the membranes, and the affinity of 3AB for 3D^pol ^and 3CD^pro ^recruits the replication complex to this new sub-cellular compartment. Within the confines of this micro-environment in the host cell cytoplasm, replication of the virus RNA genome follows a complex pathway involving the formation of intermediates – a replicative form, consisting of double stranded RNA, and a replicative intermediate, composed of a negative-strand partially hybridized to multiple nascent positive-strands [reviewed in [197]]. Briefly, viral RNA replication starts with uridylylated VPg (VPg-pU-pU)-primed synthesis of complementary negative-strand RNA molecules via the transcription of poly(A) by the RNA dependent RNA polymerase 3D^pol^. The negative-strand RNA molecules then serve as templates for the synthesis of positive-strand RNA molecules [145]. Newly synthesized positive-strand RNA molecules can serve as mRNA templates for continued translation of viral proteins or targeted as virus RNA molecules to be encapsidated in progeny poliovirions by covalent linkage of VPg to their 5' ends [135].

Encapsidation of VPg-linked positive-strand RNA molecules, a process which constitutes the final steps in the cellular life cycle of PV, appears to be linked to RNA synthesis [6] at the interface of membranous structures in the cytoplasm of infected cells [153]. To start, 3CD^pro ^cleaves the P1 precursor polypeptide, thereby giving rise to proteins VP0, VP1, and VP3, which assemble to form a protomer [195]. Five protomers then aggregate thereby generating a pentamer [156], of which twelve ultimately assemble to constitute the procapsid [88]. The VPg-linked positive-strand virus RNA may be encapsidated either by condensation of pentamers about the viral RNA [65,154] or by incorporation of the virus RNA into procapsids [88]. Cleavage of VPO into VP2 and VP4, possibly via an autocatalytic mechanism [84], finalizes virus assembly by stabilizing the capsid and thereby converting the provirion into a mature, infectious virus particle [85]. The mature virus capsid is an icosahedron composed of sixty copies each of VP1-VP4, and exhibiting five-, three-, and two-fold axes of symmetry. The outer surface of mature virus capsid is formed by capsid proteins VP1-3, while VP4 is found internally [83].

The last step in completion of the cellular life cycle, which under experimental conditions *in vitro *lasts approximately seven to eight hours, is release of mature, infectious poliovirions through lysis of the infected cell. Upon release, on the order of 1% of poliovirions will in turn initiate effective infections of permissive host cells [2].

### The poliovirus 5' Non-Translated Region (5'NTR)

Given the genetic austerity exhibited by RNA viruses, including the picornaviruses, it is surprising that they contain relatively long 5'NTRs (10% of the genome for polioviruses). These long segments of RNA, however, are packed with information displaying unique features. An important feature of the PV genomic RNA, a mRNA, that distinguishes it from most cellular mRNAs, is the absence of a 7-methyl guanosine (m^7^G) cap structure, which in cellular mRNAs interacts with the eIF4F cap-binding complex early in translation initiation of cellular proteins. In picornaviruses, the initiation of translation depends upon the internal ribosomal entry site (IRES), a novel *cis*-acting genetic element which functions as a docking site for host cell ribosomes [90,150]. Evidence for IRES-mediated, cap-independent translation of the picornavirus RNA genome emerged from experiments utilizing dicistronic RNAs harboring the IRES of encephalomyocarditis virus (EMCV) [90] or PV [150]. Jang and colleagues demonstrated that nucleotides 260–484 in the 5'NTR of EMCV were necessary for the efficient *in vitro *translation of artificial mono- and dicistronic mRNAs in nuclease-treated HeLa cell extracts and in rabbit reticulocyte lysates (RLLs) [90]. Similarly, Pelletier and Sonenberg showed that under conditions which inhibited host cell translation (in PV-infected cells), translation of the second cistron, harboring the bacterial *chloramphenicol acetyltransferase *(*CAT*) gene, mediated by the PV 5'NTR was unaffected while translation of the first cistron containing the herpes simplex virus-1 (HSV-1) *thymidine kinase *(*TK*) gene did not occur [150]. Since their discovery, IRES elements have been found in the genomes of other viruses [reviewed in [9]], including all picornaviruses (e.g., foot and mouth disease virus, FMDV [104]; hepatitis C virus, HCV [192]; and simian immunodeficiency virus, SIV [141]). IRES elements have also been discovered in cellular mRNAs of numerous organisms, including those encoding: human amyloid β A4 precursor protein [162]; fly transcription repressor hairless [116]; rat growth factor receptor [67]; and yeast transcriptional activator TFIID [87] [reviewed in [9]].

On the basis of sequence homology and comparisons of predicted structure models, the IRES elements of most picornaviruses have been classified as either type 1, exemplified by entero- and rhinoviruses, or type 2, typified by cardio- and apthoviruses [reviewed in [197]]. The two classes of IRES elements exhibit functional differences in their ability to initiate translation in cell-free translation systems such as RRLs and HeLa cell-free extracts. Type 2 IRES elements, exemplified by the EMCV IRES, initiate translation efficiently in RLLs. In contrast, type 1 IRES elements, exemplified by the PV IRES, show a deficiency in their ability to initiate translation in RLLs which is rescued by the addition of cytoplasmic extract from HeLa cells [30,46]. The difference in the ability of the type 2 IRES to initiate translation under these conditions underscores differences in host factors encountered by this class of IRES in the two systems. This in turn is suggestive of variation in the efficiency of IRES-mediated translation depending on the infected host cell, and consequently on the ability of the virus to produce pathologic changes.

In addition to the IRES domain, the 5' and 3' boundaries of which have been defined at about nt 134 and nt 556, respectively, by deletion analysis *in vitro *[86,103,133], the PV 5'NTR harbors signals important for replication of the virus RNA genome. The 5'-terminal 88 nt of the 5'NTR form a characteristic clover leaf structure, which has been shown to be an indispensable *cis*-acting element in viral RNA replication [3,144]. Additionally, the 5'NTR contains two spacer regions. One lies between the cloverleaf and the IRES (nt 89–123) and the other maps to the region between the 3' end of IRES and the initiation codon of the polyprotein (nt 640–742). The former is a sequence without a formally ascribed function. The latter has been demonstrated to be conserved in length (100–104 nt) albeit not in sequence. In line with this observation, emerging evidence indicates that the length of this spacer is important for optimal viral protein synthesis as when short open reading frames are introduced between the IRES and the initiation codon viral protein synthesis *in vitro *and, in some instances, neurovirulence are diminished [7]. Furthermore, when this spacer region between the IRES and the initiation codon is deleted, PV exhibits an *att *phenotype [180]. The function of this spacer, which is absent in the closely related rhinovirus 5'NTRs, remains a mystery. As emerging data from the Wimmer group [43] [Toyoda H, Franco D, Paul A, Wimmer E, submitted] [De Jesus N, Jiang P, Cello J, Wimmer E, unpublished] indicates, the short spacer between the cloverleaf and the IRES is loaded with genetic information essential for properties characteristic of PV.

### Interaction of trans-acting factors with the poliovirus 5'NTR

IRES-mediated translation of picornavirus RNAs involves interactions with canonical, standard eukaryotic translation initiation factors (e.g., eIF2A) as well as non-canonical, cellular *trans*-acting factors that play different roles in cellular metabolism (discussed below). Experimental techniques employed to identify host cell factors that interact with the PV 5'NTR include: RNA electrophoretic mobility shift assay, UV-mediated crosslinking of proteins to RNA, and biochemical fractionation in junction with supplementation assay. Indeed, while their abundance in cells targeted by PV remains to be characterized, a number of cellular proteins have been found to interact with the PV 5'NTR. These include: eIF2A [44]; eIF4G [161]; autoantigen La [119]; poly(rC) binding proteins 1 and 2 (PCBP1 and PCBP2) [59,144]; pyrimidine tract-binding protein (pPTB) [79,80,152]; p97/upstream of N-ras (UNR) [29]; p48/50, p38/39, and p35/36 [52,62,75,130]; p60 [75]; and nucleo-cytoplasmic SR protein 20 (Srp20) [11].

As mentioned above, the only eukaryotic translation initiation factors that have been demonstrated to interact with the PV 5'NTR are eIF2A and eIF4G. Specifically, eIF2A complexes with nt 97–182 of the PV 5'NTR [45], and deletion of a 40 amino acid region of eIF4G (642–681) substantially diminishes PV translation initiation presumably by interference with the ribosome scanning process that propels PV IRES-driven translation [161].

Among the numerous cellular proteins hypothesized to be involved in translation of the viral RNA and which have been subjected to functional analyses are the following: PCBP_1_, PCBP_2_, La, pPTB, p97/UNR [reviewed in [5]], and Srp20 [11]. PCBP_1 _and PCBP_2 _are cellular proteins each harboring three K homology (KH) RNA-binding domains. Initially termed p38, PCBP was found to interact with stem-loop IV of the PV IRES. Subsequently, PCBP was found to have affinity for stem-loop I of the 5'NTR (the cloverleaf) [59,144]. Disruption of the interaction between PCBP and stem-loop IV *in vitro *by mutations in stem-loop IV, depletion of PCBP from HeLa cell-free extracts, and injection of anti-PCBP antibodies into *Xenopus laevis *oocytes resulted in reduced translation of the viral RNA [17-19,59]. Analogously, evidence suggests that the interaction between PCBP and the cloverleaf (specifically stem-loop B) is also necessary for efficient translation of the virus RNA [60,178]. Stem-loop D of the cloverleaf RNA binds the viral protein 3CD (and, very poorly, 3C^pro^). The cloverleaf, PCBP, and 3CD for a ternary complex that is essential for initiation of plus-strand RNA synthesis [3,4,48,168]. It has been hypothesized to be involved in a switch mechanism governing use of the viral RNA as either a template for translation or replication. Binding of 3CD to a complex formed by the cloverleaf RNA and PCBP inhibits translation in a cell-free extract and is hypothesized to promote replication, thereby providing a mechanism to ensure an adequate balance between these two processes. Incompatibility of the cloverleaf RNA with the viral 3CD, as in the context of chimeras, would be expected to result in decreased virus viability. Indeed, while it has been shown that a virus containing the 5'NTR of CB3 (nt 1–625) and remaining parts of the genome from PV1(M) was viable [92], a virus containing the 5'NTR of human rhinovirus 14 (HRV14) and the remainder parts of the genome from PV3, exhibited a lethal phenotype, because the PV 3CD was unable to interact effectively with the HRV14 stem-loop D [168]. In the latter, the virus was rescued by insertion of two nucleotides into stem-loop D (CUAC_60_GG_61_) of the HRV14 cloverleaf [167].

The nuclear protein La is an autoantigen targeted by antibodies produced by patients with autoimmune disorders such as systemic lupus erythematous and Sjogren's syndrome. Normally, it functions in termination of RNA polymerase III transcription [68,69]. First characterized as HeLa cell protein p52, La is found in HeLa cell-free extracts but not in RLLs. Supplementation of RLLs with La has been demonstrated to stimulate translation of PV RNA [120].

The nuclear protein polypyrimidine tract-binding protein (PTB), also known as hnRNP 1, which plays a role in alternative splicing of the cellular pre-mRNA [61,66,143], associates with three sites within the PV 5'NTR (nt 70–286, nt 443–539, and nt 630–730) as determined by UV-crosslinking [80]. An attenuating mutation, C_472_U, reduced the affinity of the PV 5'NTR to pPTB in neuroblastoma cells (SH-SY5Y) without disrupting this interaction in HeLa cells [74]. nPTB, a neuronal-cell specific homologue of PTB, was later described to bind less efficiently to the PV IRES in the presence of the C_472_U attenuating mutation [73].

The cytoplasmic RNA-binding protein UNR was originally identified as p97 in HeLa cells and lacking in RLLs. Studies in which endogenous expression of the *unr *gene was disrupted by homologous recombination, transient expression of UNR effectively reestablished efficient translation by human rhinovirus and PV IRESs [28].

Lastly, Srp20 is a member of the SR family of splicing regulators. Recently, it has been found to interact with PBCP_2 _[11], a cellular RNA-binding protein that (as discussed above) binds to sequences within the PV IRES and is necessary for translation of the viral RNA. Bedard and colleagues [11] have shown that PV translation is inhibited by depletion of Srp20 in HeLa cell extracts and diminished by down-regulation of Srp20 protein levels by RNA interference *in vivo*. Whether Srp20 interacts directly with IRES sequences was not determined.

### Poliovirus pathogenesis

PV tropism is limited to humans and non-human primates. In its natural host, PV transmits via the fecal-oral route. To date, the specific sites and cell types in which the virus initially replicates following entry into the host remain enigmatic. Nevertheless, the ability to isolate virus from the lymphatic tissues of the gastrointestinal tract, including the tonsils, Peyer's patches of the ileum, and mesenteric lymph nodes [24,25,106,173,174], as well as the feces [106,174], prior to the onset of illness suggests susceptible cells in these tissues may be sites of primary replication. Following initial replication of the virus in susceptible cells of the pharynx and gastrointestinal tract, in the majority of infected individuals a minor, transient viremia, but no neurologic complications, will develop. As the infection progresses, the virus will spread further to other sites of the reticuloendothelial system. Consequently, the great majority of PV infections, nearly 95%, including almost all infections in which a minor viremia develops, are 'innaparent' or asymptomatic. In 4–8% of infected individuals that develop primary viremia, a secondary, major viremia often associated with a 'minor, non-specific illness' will ensue. Also known as abortive poliomyelitis, the clinical manifestations of this 'minor, non-specific illness' include many signs and symptoms generally associated with other viral illnesses: (a) an upper respiratory infection, characterized by sore throat and fever; (b) a gastrointestinal illness, presenting with nausea, vomiting, abdominal discomfort, and constipation or (infrequently) diarrhea; and/or (c) an illness mimicking influenza, marked by headache, myalgia, and generalized malaise [24,106,174]. In turn, a minute segment of infected individuals that experience major viremia will progress to develop signs and symptoms indicating PV invasion of the CNS, as characterized by non-paralytic aseptic meningitis or paralytic poliomyelitis. Non-paralytic aseptic meningitis occurs in 1–2% of PV infections and is associated with rigidity of the neck, back, and lower limbs as well as an augmented number of leukocytes (10–200 cell/mm^3^) and slightly above-normal protein levels (40–50 mg/dL) in the cerebrospinal fluid (CSF) [35]. Paralytic poliomyelitis occurs in 0.1–1% of all PV infections, depending on the offending serotype [132]. Based on the specific manifestation, paralytic poliomyelitis without apparent affect in sensation or cognition is classified as either: (i) spinal poliomyelitis, characterized by acute flaccid paralysis secondary to selective destruction of spinal motor neurons and subsequent dennervation of the associated skeletal musculature; (ii) bulbar poliomyelitis, presenting with paralysis of respiratory muscles following attack of neurons in the brain stem that control breathing; and (iii) bulbospinal poliomyelitis, exhibiting effects on both the brain stem and spinal cord [26,35]. Among cases of paralytic poliomyelitis, it is estimated that fatalities result in 2–5% of children and 15–30% of adults, numbers which are drastically increased in cases featuring bulbar paralysis [35].

Isolation of PV from the CSF is diagnostic but seldom achieved [35]. Additionally, the precise mechanism(s) of PV invasion of the CNS is not well understood. Three hypotheses for mechanisms utilized by the virus to gain entry into the CNS have been proposed: (1) the virus invades the CNS by retrograde axonal transport [71,138,139]; (2) the virus crosses the blood-brain barrier (BBB), presumably independent of the presence of the cellular receptor for PV, CD155 [200]; and (3) the virus is imported into the CNS by infected macrophages – the Trojan horse mechanism [51,57]. In support of the theory of CNS invasion due to permeation of the BBB, Yang and colleagues found that PV accumulated in the CNS of *CD155 *transgenic (tg) mice at a constant rate that was markedly higher than the accumulation rate for albumin, which is not believed to cross the BBB [200]. Earlier, Blinzinger *et al., *had interpreted their own finding of PV particles in endothelial cells forming part of the BBB to indicate that the virus breached the CNS through its vasculature [15]. Following this line of thought, evidence for entry of PV into the CNS via infected macrophages is largely circumstantial, emerging from observations that PV replicates in macrophages expressing CD155 [51,57] and that macrophages infected with Visna virus [151] and human immunodeficiency virus (HIV) [54] traverse the BBB.

However, experimental evidence from studies in non-human primates [22,23] and *CD155 *tg mice [62,138,165] supports the hypothesis of CNS invasion mediated by retrograde axonal transport along peripheral nerves. The observations that paralysis of the injected limb can be prevented by transection of the nerve linking the site of injection to the spinal cord, and that skeletal muscle injury concurrent with PV infection predisposes to paralysis initially localizing to the afflicted limb (as observed in phenomena denoted provocation poliomyelitis and iatrogenic poliomyelitis) [71,131], strongly suggest a neural pathway for PV entry into the CNS. Specially strong evidence supporting a neural pathway of CNS invasion emerged from a study published by Ohka *et al*., in which the authors reported recovery of intact 160S virion particles in the sciatic nerve of *CD155 *tg mice transected at various intervals following intramuscular inoculation with PV, an observation suggesting a role for fast retrograde axonal transport driving poliovirions along peripheral nerves to the spinal cord, where the cell bodies of motor neurons targeted by the virus reside [138]. This observation supported early reports of the presence of PV in axons during experimental poliomyelitis [20,55].

### Poliovirus vaccines

Prior to the 20^th ^century, virtually all children were infected with PV while still protected by maternal antibodies. In the 1900s, following the industrial revolution of the late 18^th ^and early 19^th ^centuries, improved sanitation practices led to an increase in the age at which children first encountered the virus, such that at exposure children were no longer protected by maternal antibodies [132]. Consequently, epidemics of poliomyelitis surfaced [35].

In the mid-20^th ^century, in efforts to combat the ever growing epidemics of poliomyelitis ravaging the United States, research focused on the design of vaccines as a means of halting transmission. The first vaccine to be produced was the inactivated (or "killed") PV vaccine (IPV) by Jonas Salk on April 12, 1955. In producing IPV, all three PV serotypes were (and continue to be) grown *in vitro *in African green monkey kidney (Vero) cells and inactivated by formaldehyde. IPV was shown to effectively immunize and protect against poliomyelitis [35].

A second vaccine which was demonstrated to be both safe and effective was the oral (or "live") PV vaccine (OPV) developed by Albert Sabin in 1963. In truth, testing of the vaccine began in 1957 under the auspices of the WHO, but it was not until 1961 that the United States Public Health Service endorsed OPV, then only produced in the monovalent form. Trivalent OPV (or simply "OPV" as will be referred to henceforth) became available in 1963 and, owing to its unique ability to produce unmatched gastrointestinal immunity, thereby preventing infection with *wt *virus, soon became the preferred PV vaccine in the United States and many other countries. OPV is composed of *att *strains of all three PV serotypes, grown *in vitro *in Vero cells, in a 10:1:3 ratio of types 1:2:3, respectively [35].

The *att *strains comprising OPV were generated by serial passage of *wt *strains at high multiplicity of infection (MOI) in a series of hosts ranging from cells derived from a variety of sources including monkey testis, kidney, and skin to live monkeys [124], accompanied by selection of variants following experimental bottlenecking events such as single-plaque cloning and limiting dilution. The desirable characteristics of selected variants were: (i) ability to replicate effectively in the gastrointestinal tract; (ii) defectiveness in the ability to invade or replicate within the CNS; and (iii) genetic stability so as to withstand the pressures of replication within the human host without reversion to a neurovirulent phenotype. These qualities were those present in variants which came to be the Sabin vaccine strains.

Years later, comparison of the nucleotide sequences of the *att *Sabin strains and their neurovirulent parental strains revealed a series of mutations, some of which were subsequently found to be responsible for the *att *phenotypes of the Sabin strains. PV type 1 (Sabin) [PV1(S)] harbored 7 nucleotide substitutions localizing to the 5'NTR, 21 amino acid alterations within the polyprotein, and 2 nucleotide substitutions within the 3'NTR [157]. PV type 3 (Sabin) [PV3(S)] contained 2 nucleotide substitutions in the 5'NTR, 4 amino acid changes within the polyprotein, and a single nucleotide deletion within the 3'NTR [216]. Lastly, PV type 2 (Sabin) [PV2(S)] exhibited a single nucleotide substitution within the 5'NTR as well as one amino acid change within the polyprotein [115,147,164]. Subsequent sequence analysis of revertants with regained neurovirulence indicated that mutations mapping to the 5'NTR specified the *att *phenotype of the three Sabin strains. Attenuating point mutations within the 5'NTR of the Sabin vaccine strains (nt 480, 481, and 472 in serotypes 1, 2, and 3, respectively) localize to the IRES (domain V) (Fig. 2) and their presence has been linked to deficiencies in viral replication in the CNS and in neuroblastoma cells [106,107] as well as reductions in translation of the viral mRNAs as compared to *wt *sequences [184-186].

Moreover, all Sabin strains exhibit *ts *phenotypes, which map to the 5'NTR mutation (for all 3 types) [94,114,118], to the capsid precursor (for all 3 types) [27,107,114,142], as well as to the 3D^pol ^coding sequence (for type 1) [27,39,118,146,187,191]. The *ts *phenotype is thought to be the most important trait of the vaccines to confer attenuation.

### Poliovirus eradication and evolution

Thanks in part to the effectiveness and ease of administration of OPV as well as to the efforts of public health officials in the United States, the transmission of *wt *PV was halted by 1979, less than 20 years since introduction of OPV [35]. Indeed, OPV was the weapon of choice in the fight against vaccine-preventable poliomyelitis of the Pan American Health Organization (PAHO) under the leadership of Ciro de Quadros, M.D., M.P.H. By transforming vaccines and immunization against PV into a top priority of governments, vaccine producers, and public health experts, de Quadros was able to institute teams to further his cause at the Ministry of Health in nearly every country in the Americas. In 1985, PAHO announced its goal to eradicate *wt *PV in the Western Hemisphere by 1990. The target date was met. The last case of *wt *PV-induced paralytic poliomyelitis was documented in Peru in 1991. Three years later, in 1994, the International Commission for the Certification of Poliomyelitis Eradication announced that transmission of *wt *PV in the Americas had been discontinued.

Decades prior, while the United States was actively attempting to halt transmission of *wt *PV by vaccination with OPV, the WHO was trying to finalize the eradication of another highly infectious agent – smallpox. By 1967, programs to eradicate smallpox had proven successful in many regions of the globe, including Western Europe, North America, and Japan. In 1967, in line with recommendations made by a WHO Expert Committee on Smallpox 3 years earlier to vaccinate the entire world's population as a means of furthering efforts to eradicate the variola virus, the WHO introduced the Intensified Smallpox Eradication Program. The mass vaccination strategy employed to eradicate an agent, estimated to have caused 10–15 million cases of smallpox as early as 1967, eventually paid off. The last recorded case of smallpox occurred in Somalia in 1979. In 1980, the 33^rd ^World Health Assembly announced the first successful eradication of a major human disease – smallpox [56].

In 1988, the WHO envisioned the eradication of yet another agent causing major human disease (i.e., PV) by launching a global campaign to eradicate *wt *PV by the year 2000. Of the two available polio vaccines, the Sabin OPV was chosen to further the planned eradication efforts. Two characteristics of OPV propelled it for selection by the WHO as the instrument of choice in the Global Polio Eradication Initiative: (1) its effectiveness in producing gastrointestinal immunity; and (2) the fact that no special instrumentation (i.e., needles) was required for its administration.

Undoubtedly, the use of OPV in mass vaccinations has resulted in dramatic reductions in the number of cases of poliomyelitis due to infections with *wt *PV from an estimated 350,000 in over 125 endemic countries in 1988 to just 1,874 in 2006. But perhaps counter-intuitively, the use of OPV now poses enormous challenges to this endeavor. A major flaw of OPV is that it is genetically unstable, a characteristic that makes it particularly susceptible to evolve into circulating vaccine-derived polioviruses (cVDPV), exhibiting *wt *PV-like properties, including neurovirulence. The Centers for Disease Control (CDC) estimates that in the United States, while OPV was being used, one case of vaccine-associated paralytic poliomyelitis (VAPP) emerged for every 2 to 3 million doses of OPV administered, which accounted for 8 to 10 cases of VAPP in this country per year. In fact, in the United States, the vast majority (95%) of cases of paralytic poliomyelitis documented between 1980 and 1999 resulted from cVDPV-induced VAPP [35]. In 1996, in order to reduce the incidence of VAPP among vaccine recipients, the United States Advisory Committee on Immunization Practices (ACIP) recommended the increased use of IPV by replacing the first two vaccine doses of the immunization schedule with IPV as opposed to OPV. While the risk of VAPP was reduced among vaccine recipients, the equivalent reduction in risk did not translate for non-immune contacts of vaccine recipients. With this in mind, in 1999, the ACIP recommended that starting in the year 2000 use of OPV be discontinued and that IPV be used exclusively in the United States.

Specifically, genetic changes that would endow OPV with *wt *PV phenotypes, transforming *att *vaccine strains into cVDPV with the ability to cause VAPP among vaccine recipients and/or their close contacts, could take the form of reversions of known attenuating mutations and recombination, whether inter- or hetero-typic [2]. Certainly, as discussed above, comparisons of the nucleotide sequences of *wt *PV strains with revertants of the *att *Sabin OPV strains were key in identifying the determinants of attenuation. But perhaps just as important in the genesis of cVDPV is the possibility of recombination. Admittedly, recombination among human enteroviruses has been hypothesized to be a common occurrence in nature.

In fact, recombination between RNA viruses, a process originally considered highly unlikely, was shown first with polioviruses [82]. Later, Agol and his colleagues provided evidence that recombination can also occur between different serotypes of PV [170,189]. Significantly, the 3 Sabin vaccine strains have been shown to undergo rampant intertypic recombination in vaccine recipients [17,33,37,42,63,64,93,105,111,117,148]. Finally, recombination between polioviruses was shown to occur in a cell-free HeLa cell extract [49].

It has been speculated that recombination may serve as a mechanism to augment the potential of viruses to adapt and evolve. The evolution of OPV into highly diverged cVDPV, via recombination between PV and other closely related enteroviruses, in inadequately immunized populations is a very real concern [8,95,112,171,177]. For the most part, recombinants between the Sabin vaccine strains and other human enteroviruses exhibit crossover points within the non-structural region of the genome, such that the PV 5'NTR and structural P1 regions are retained. Consequently, of particular concern are recombinations that results in viruses which have lost the *att *phenotype of the parental vaccine strains and gained phenotypic characteristics that would make them indistinguishable from *wt *PV, thereby acquiring the ability to cause poliomyelitis.

Recently, in the current climate of attempting to eradicate PV, the possibility of genetic exchanges between the Sabin OPV strains and closely related viruses has come into the limelight. Indeed the propensity of OPV strains to recombine in recipients of the vaccine has been documented in numerous outbreaks of VAPP secondary to the unchecked circulation of cVDPV in poorly immunized communities [8,95,112,171,177]. Moreover, the possibility exists that, as previously hypothesized [[72,166]; Jiang P, Faase JAJ, Toyoda H, Paul A, Gorbalenya AE, Wimmer E, unpublished], in a world free of PV and anti-PV antibodies as envisioned by the WHO, viruses closely related to the polioviruses such as the C-cluster coxsackie A viruses (e.g., CAV20) may fill the niche left vacant by the polioviruses. Could C-cluster coxsackie A viruses evolve to utilize CD155 as a cellular receptor, thereby completely altering the disease syndromes with which they would be associated? If only the structural region of C-cluster coxsackie A viruses evolved to recognize the PV cellular receptor while maintaining the rest of the genome unchanged, would such a virus replicate in the same cell types and to the same levels as *wt *PV or would the C-cluster coxsackie A virus-specific genome segments impose cell-internal restrictions on viral replication? If simply the presence of C-cluster coxsackie A virus-derived genome segments results in restricted viral replication, which particular genome segments are accountable for such phenotypic differences? Moreover, would attenuating mutations in the PV genome translate into attenuating mutations in viruses that result from the recombination of PV with a closely-related yet non-neurovirulent C-cluster coxsackievirus? These are precisely some of the questions currently under investigation in the Wimmer laboratory.

### The biochemical synthesis of poliovirus

Despite the undeniable success of the Global Polio Eradication Initiate in the nineteen years since its introduction, characteristics inherent to OPV, logistical obstacles in ensuring 100% vaccination, as well as the realization that *de novo *synthesis of viruses is a possibility, have brought into question the feasibility of the control of poliomyelitis by means of the total eradication of *wt *PV. Current recommendations by the WHO include the cessation of OPV vaccination 3 years following the last reported case of poliomyelitis due to infection with *wt *PV. In time, cessation of vaccination would inevitably result in lost of herd immunity, which in turn generates an ever increasing pool of susceptible individuals to the same agent against which immunization was originally targeted and to one similar that is evolving to replace it. In such discussions, an important consideration remains: can a virus that can be synthesized ever truly be considered eradicated?

Evidence for the ability to chemically synthesize a virus (i.e., PV) first emerged from a study published by Eckard Wimmer's group in 2002 [34], which described the *de novo *biochemical synthesis of infectious PV by utilizing as instruction only the published nucleotide sequence of the genome. The initial step in the scheme to synthesize PV1(M) consisted of generating a complete complementary DNA (cDNA) copy of the virus genome bearing a phage T7 RNA polymerase promoter at the 5' end. This endeavor was accomplished by a laborious process in which: (i) overlapping segments of 400 to 600 base pairs (bp) were synthesized by piecing together purified oligonucleotides (approximately 69 nt in length) of plus and minus polarity with overlapping complementary sequences at the ends, followed by ligation of the segments into a plasmid vector; (ii) cloned segments were sequenced to pinpoint segments with correct sequences and those containing only a small number of mutations that could be corrected either by sub-cloning or by site-directed mutagenesis; (iii) cloned segments were sequentially joined to generate three large DNA fragments 3026, 1895, and 2682 bp in length; and (iv) combining the three DNA fragments to produce the full-length sPV1(M) cDNA. To ensure the *wt *sequence of PV1(M) could be distinguished from that of sPV1(M), in generating the sPV1(M) cDNA, 27 nucleotide substitutions were engineered as markers in the sPV1(M) cDNA. Next, the T7 promoter-containing sPV1(M) cDNA was transcribed *in vitro *with T7 RNA polymerase to yield highly infectious virus RNA, which was equivalent in length to virion RNA. The presence of all genetic markers engineered into the sPV1(M) cDNA was established by restriction enzyme digest analysis of products of reverse transcriptase-polymerase chain reaction (RT-PCR) in which virus RNA isolated from sPV1(M)-infected HeLa cells was used as template. *De novo *synthesis of PV from transcript RNA derived from sPV1(M) cDNA in a cell-free extract of uninfected HeLa cells, as previously described for *wt *PV1(M) [146], was confirmed with the yield of end products of proteolytic processing of the virus polyprotein as well as the production of infectious virus. Comparison of virus-specific proteins generated by incubation of sPV1(M) cDNA-derived transcript RNA with an S3 cytoplasmic extract of HeLa cells with the corresponding proteins derived from *wt *PV1(M) cDNA-derived transcript RNA validated the products of *in vitro *translation as PV-specific proteins. The production of infectious virus by incubation of sPV1(M) cDNA-derived transcript RNA with an S3 cytoplasmic extract of HeLa cells was ascertained by analysis of plaque formation on HeLa cell monolayers on which aliquots of the transcript RNA-containing cytoplasmic extract had been incubated. The ability of CD155-specific monoclonal antibody (mAb) D171 to block infection of HeLa cells by sPV1(M) was verified by the observation that incubation of HeLa cells with mAb D171 prior to addition of sPV1(M) entirely voided the virus' plaque forming ability. Lastly, to characterize the disease-inducing potential of sPV1(M), the neurovirulence phenotype of this virus was examined in *CD155 *tg mice. Following intracerebral (i.c.) inoculation with sPV1(M), adult *CD155 *tg mice often developed neurological signs characteristic of poliomyelitis, including flaccid paralysis and even death. The inoculum required to produce paralysis and/or death in half the mice inoculated (PLD_50_) was markedly increased over that required to produce the same signs of disease with *wt *PV1(M). The degree of attenuation of sPV1(M) compared to the parental PV1(M) was rather unanticipated. All nucleotide substitutions engineered into the sPV1(M) ORF, with the exception of XmaI and StuI restriction sites generated in the 5'NTR and 2B regions, produced silent mutations. But the changes in the 2B coding region had previously been demonstrated not to influence virus replication *in vitro *[126,198]. Hence, when the findings were first published, the authors attributed the *att *phenotype of sPV1(M) in *CD155 *tg mice to the silent mutations and then unidentified mechanisms [34].

In a subsequent study [43], the authors set forth to determine what aspect of the sPV1(M) genome was responsible for the phenotypic changes observed in comparisons with *wt *PV1(M). In collaboration with others the author of this review showed that a single nucleotide substitution (A_103_G) mapping to the spacer region between the cloverleaf and the IRES within the 5'NTR determines the *att *phenotype of sPV1(M).

In our quest to determine what alterations in the genotype of sPV1(M) resulted in the observed neurophenotypic changes, two strategies were employed: (1) exchange of genomic segments between sPV1(M) and *wt *PV1(M) followed by analysis of neurovirulence *in vivo*; and (2) sequence analysis of viruses recovered from the spinal cords ofsPV1(M)-inoculated *CD155 *tg mice that had succumbed to infection in concert with comparison of these sequences with that of sPV1(M) virus that constituted the inoculum. In all instances, we identified a change at one locus in the sequences of recovered viruses. Analyses of the *in vitro *phenotypes in tissue culture as well as the *in vivo *phenotypes in *CD155 *tg mice of a series of PV variants revealed the critical nucleotide in determining two important characteristics of sPV1(M): (i) an *att *neurophenotype in adult *CD155 *tg mice; and (ii) a *ts *phenotype in neuronal cells of human origin.

Considering that the nucleotide we identified as an important determinant of the replicative phenotypes of PV *in vivo *as well as *in vitro *(A_103_) is highly conserved among polioviruses and human C-cluster coxsackie A viruses and that, in evolution, conservation often equates with functional importance, we have continued our analysis of this locus to studying the effect of mutating this nucleotide (A_103_G) in CAV20 – one of three human C-cluster enteroviruses exhibiting the highest degree of sequence homology to the polioviruses [De Jesus N, Jiang P, Cello J, Wimmer E, unpublished].

## Conclusion

Whether accidentally or not, over centuries poliovirus has evolved to specifically target alpha motor neurons in the spinal cord of its human host, thereby causing acute flaccid paralysis, the characteristic sign of paralytic poliomyelitis. Fortunately, since its inception in 1988, the WHO's Global Polio Eradication Initiative, along with great economic and intellectual efforts, has served to greatly reduce the number of documented cases of poliomyelitis worldwide. Nonetheless, the ultimate goal of halting poliovirus transmission as a means of eradicating poliomyelitis has proven rather elusive. In light of this realization and considering the possibility that a virus that can be synthesized may never truly be considered eradicated, it is imperative that new strategies to combat poliovirus be considered, whether these be in the form of the development of new vaccines and/or anti-viral drugs. In this endeavor it is important that aspects of the pathogenesis of this virus, such as interactions with host factors that play roles in replication and/or translation of the viral genome be identified, as well as sites of primary replication and the mechanism(s) of CNS invasion be more clearly elucidated. For it is only by understanding the intricacies of the life cycle of this pathogen within the human host that we will be able to more effectively develop new treatment modalities.

## Competing interests

The author declares that she has no competing interests.
